# MicroRNAs as Diagnostic Biomarkers and Predictors of Antidepressant Response in Major Depressive Disorder: A Systematic Review

**DOI:** 10.7759/cureus.56910

**Published:** 2024-03-25

**Authors:** Beatriz A Carneiro, Lívia N Franco Guerreiro-Costa, Daniel Lins-Silva, Daniela Faria Guimaraes, Lucca S Souza, Gustavo C Leal, Ana Teresa Caliman-Fontes, Graziele Beanes, Ryan Dos S Costa, Lucas C Quarantini

**Affiliations:** 1 Medicine, Laboratório de Neuropsicofarmacologia, Serviço de Psiquiatria do Hospital Universitário Professor Edgard Santos, Universidade Federal da Bahia, Salvador, BRA; 2 Medicine, Universidade Federal da Bahia, Salvador, BRA; 3 Medicine, Programa de Pós-Graduação em Medicina e Saúde, Faculdade de Medicina da Bahia, Universidade Federal da Bahia, Salvador, BRA; 4 Medicine, Laboratório de Imunofarmacologia e Biologia Molecular, Instituto de Ciências da Saúde, Universidade Federal da Bahia, Salvador, BRA; 5 Psychiatry, Universidade Federal da Bahia, Salvador, BRA

**Keywords:** micrornas, biomarkers, antidepressive agents, major, depressive disorder

## Abstract

Despite the hardships of major depressive disorder (MDD), biomarkers for the diagnosis and pharmacological management of this condition are lacking. MicroRNAs are epigenetic mechanisms that could provide promising MDD biomarkers. Our aim was to summarize the findings and provide validation for the selection and use of specific microRNAs as biomarkers in the diagnosis and treatment of MDD. A systematic review was conducted using the PubMed/Medline, Cochrane, PsycINFO, Embase, and LILACS databases from March 2022 to November 2023, with clusters of terms based on “microRNA” and “antidepressant”. Studies involving human subjects, animal models, and cell cultures were included, whereas those that evaluated herbal medicines, non-pharmacological therapies, or epigenetic mechanisms other than miRNA were excluded. The review revealed differences in the expression of various microRNAs when considering the time of assessment (before or after antidepressant treatment) and the population studied. However, due to the heterogeneity of the microRNAs investigated, the limited size of the samples, and the wide variety of antidepressants used, few conclusions could be made. Despite the observed heterogeneity, the following microRNAs were determined to be important factors in MDD and the antidepressant response: mir-1202, mir-135, mir-124, and mir-16. The findings indicate the potential for the use of microRNAs as biomarkers for the diagnosis and treatment of MDD; however, more homogeneous studies are needed.

## Introduction and background

Major depressive disorder (MDD) is a debilitating disorder that has a prevalence rate of greater than 10% [1,2] and a remission rate after conventional treatment of approximately 50% [3]. Genetic variants are involved in the pathophysiology of mental disorders and possibly in the treatment response, with a study reporting that 42% of the variation in outcomes after treatment can be attributed to genetic factors [4]. Therefore, a greater understanding of the genetics involved in MDD can help optimize pharmacological treatments by increasing the accuracy of drug response and tolerability predictions.

Our current understanding of the molecular pathophysiology of MDD is limited, although studies have associated MDD with genetic polymorphisms, epigenetic mechanisms, and synaptic plasticity alterations [5-7]. Some genetic features are inherited; however, most are modified during prenatal development and throughout the lifetime by various environmental factors, including food and drug exposure [8]. Three epigenetic mechanisms function in the modulation of gene expression [9]. Two of these change the DNA structure without affecting its sequence (DNA methylation and histone acetylation) and the third modulates gene expression at the post-transcriptional level (miRNAs) [9].

MicroRNAs (miRNAs) are small non-coding RNAs that influence and regulate gene expression, thereby acting as epigenetic modulators [10]. By binding to specific genes, these miRNAs affect neural plasticity and brain function by inhibiting translation or degrading mRNA [10]. Previous animal studies found that stress, which is a risk factor for MDD, interfered with miRNA expression in rodent brains [11,12]. This finding supports the involvement of miRNAs in the etiopathogenesis of MDD, highlighting a possible role for these molecules in the diagnosis of psychiatric diseases. Furthermore, other studies have identified strong associations between certain miRNAs and antidepressant (AD) responses, including miR-1202, miR-124, miR-135a, miR-145, and miR-20b, which are promising response biomarker candidates [13]. Lopez et al. [14] demonstrated that miR-146a-5p, miR-146b-5p, miR-425-3p, and miR-24-3p levels decreased following AD treatment. However, the expression of miRNAs between different body components and the relationship between the peripheral and central levels of this biomolecule are inconsistent.

The pathophysiology of MDD is a promising ongoing topic of research. However, we believe that the contribution of miRNAs is under-explored, despite the considerable interest in obtaining biological biomarkers for MDD diagnosis and AD treatment response [15-18]. The purpose of our review was to investigate changes in miRNA expression before and after pharmacological treatment for MDD.

## Review

Methods

We conducted a systematic review of the literature following the recommendations of the Cochrane Handbook [19] and preferred reporting items for systematic reviews and meta-analyses (PRISMA) guidelines [20]. The review was registered in the PROSPERO database (CRD42021265268).

Literature Search and Study Selection

From March 2022 through November 2023, we searched the PubMed/Medline, Cochrane, PsycINFO, Embase, and LILACS databases using the following terms: (micrornas OR microrna OR mirnas OR micro rna) AND (antidepressive agents OR antidepressive drugs OR antidepressive agent OR antidepressive OR antidepressants OR antidepressant OR antidepressant drugs OR antidepressant drug OR antidepressive treatment). No limits to publication years were added. The articles were required to be published in English and to involve humans, animals, or cell cultures. The titles and abstracts of the retrieved items were independently screened by four reviewers (LG, BC, DH, LS), and the two main reviewers (LG, BC) then independently assessed the full texts of the selected studies.

Eligibility Criteria

The strategic question that guided the manual search and the inclusion and exclusion criteria was as follows: “What are the levels of miRNA expression before and after AD treatment?” The inclusion criteria were (1) original studies only; (2) human subjects aged 18 or over with a clinical diagnosis of MDD and undergoing treatment with ADs; and (3) animal models and cell cultures with miRNA assessments after treatment with ADs. For each study, we prepared a standardized spreadsheet that presented the following information: i) authors and publication year, ii) publication journal, iii) population studied, iv) treatment conducted, v) instrument used for diagnosis and stratification of MDD, vi) study design, vii) miRNA extraction site, viii) follow-up time, and ix) miRNA expression before and after intervention. We excluded studies that evaluated herbal medicines, non-pharmacological therapies, or epigenetic mechanisms other than miRNAs.

Risk of Bias Assessment

The quality of the study was evaluated by two reviewers (DF, DH) using the strengthening the reporting of observational studies in epidemiology (STROBE) [21] and the systematic review center for laboratory animal experimentation (SYRCLE) [22] tools for human and animal studies, respectively (Tables 1-2). We did not assess the quality of the cell culture studies because no standardized tool exists for this purpose. Discrepancies between the two reviewers were resolved through discussions until a consensus was reached.

**Table 1:** Strengthening the Reporting of Observational Studies in Epidemiology (STROBE) for human studies

Items	Belzeaux et al., 2017 [13]	Lopez et al., 2017 [14]	Bocchio-Chiavetto et al., 2013 [23]	Homorogan et al., 2021 [24]	Issler et al., 2014 [25]
Title and abstract	X	X	X	X	
Introduction					
Background/rationale	X	X	X	X	X
Objectives	X	X	X	X	X
Methods					
Study design	X	X	X	X	X
Setting	X	X	X	X	
Participants	X	X	X	X	X
Variables	X	X	X	X	X
Data sources/measurement	X	X	X	X	X
Bias		X			
Study size					
Quantitative variables	X	X	X	X	X
Statistical methods	X	X	X	X	X
Results					
Participants		X			
Descriptive data	X		X	X	
Outcome data	X	X	X	X	X
Main results	X	X	X	X	X
Other analyses	X	X	X	X	X
Discussion					
Key results	X	X	X	X	X
Limitations	X			X	
Interpretation	X	X	X	X	X
Generalisability	X	X	X	X	X
Other information					
Funding	X	X	X	X	X
Total	19	18	17	19	15
Items	Enatescu et al., 2016 [26]	Saeedi et al., 2021 [27]	Fang et al., 2018 [28]	Wang et al., 2018 [29]	Kuang et al., 2018 [30]
Title and abstract	X		X	X	X
Introduction					
Background/rationale	X	X	X	X	X
Objectives	X	X	X	X	X
Methods					
Study design	X	X	X	X	X
Setting	X	X	X		X
Participants	X	X	X	X	X
Variables	X	X	X	X	X
Data sources/measurement	X	X	X	X	X
Bias					
Study size	X		X		X
Quantitative variables	X	X	X	X	X
Statistical methods	X		X	X	X
Results					
Participants	X				
Descriptive data	X	X	X	X	X
Outcome data	X	X	X	X	X
Main results	X	X	X	X	X
Other analyses	X	X	X	X	X
Discussion					
Key results	X	X	X	X	X
Limitations	X		X	X	X
Interpretation	X	X	X	X	X
Generalisability	X	X			
Other information					
Funding		X	X	X	X
Total	18	15	19	16	19
Items	Lin et al., 2018 [31]	Fiori et al., 2017 [32]	Kato et al., 2022 [33]	Kim et al., 2019 [34]	Marshe et al., 2020 [35]
Title and abstract	X	X	X	X	X
Introduction					
Background/rationale	X	X	X	X	X
Objectives	X	X	X	X	X
Methods					
Study design	X	X	X	X	X
Setting	X			X	X
Participants	X		X		X
Variables	X	X	X	X	X
Data sources/measurement	X	X	X	X	X
Bias			X		
Study size	X				X
Quantitative variables	X	X	X	X	X
Statistical methods	X		X	X	X
Results					
Participants			X		
Descriptive data	X		X		X
Outcome data	X	X	X	X	X
Main results	X	X	X	X	X
Other analyses		X	X	X	X
Discussion					
Key results	X	X	X	X	X
Limitations	X		X	X	X
Interpretation	X	X	X	X	X
Generalisability			X	X	X
Other information					
Funding	X	X	X	X	X
Total	18	13	20	17	20
Items	Gururajan et al., 2016 [36]	Belzeaux et al., 2012 [37]	Qiao-li et al., 2014 [38]	He et al., 2016 [39]	Hung et al., 2019 [40]
Title and abstract	X		X	X	X
Introduction					
Background/rationale	X	X	X	X	X
Objectives	X	X	X	X	X
Methods					
Study design	X	X	X	X	X
Setting	X		X	X	X
Participants	X	X	X	X	X
Variables	X	X	X	X	X
Data sources/measurement	X	X	X	X	X
Bias			X		
Study size					X
Quantitative variables	X	X	X	X	X
Statistical methods	X	X	X	X	X
Results					
Participants			X		X
Descriptive data	X	X	X	X	X
Outcome data	X	X	X	X	X
Main results	X	X	X	X	X
Other analyses	X	X	X	X	X
Discussion					
Key results	X	X	X	X	X
Limitations	X	X		X	X
Interpretation	X	X	X	X	X
Generalisability			X		X
Other information					
Funding	X	X		X	X
Total	18	16	19	16	18
Items	Qi et al., 2020 [41]	Hung et al., 2021 [42]	Liu et al., 2021 [43]	Song et al., 2022 [44]	
Title and abstract	X				
Introduction					
Background/rationale	X	X	X	X	
Objectives	X	X	X	X	
Methods					
Study design	X	X	X	X	
Setting	X	X	X	X	
Participants	X	X	X	X	
Variables	X	X	X	X	
Data sources/measurement	X	X	X	X	
Bias					
Study size					
Quantitative variables	X	X	X	X	
Statistical methods	X	X	X	X	
Results					
Participants					
Descriptive data	X	X	X	X	
Outcome data	X	X	X	X	
Main results	X	X	X	X	
Other analyses		X	X	X	
Discussion					
Key results	X	X	X	X	
Limitations		X	X	X	
Interpretation	X	X	X	X	
Generalisability		X	X		
Other information					
Funding	X	X	X	X	
Total	16	18	18	17	

Items	Emami et al., 2023 [45]	Wang et al., 2023 [46]	Funatsuki et al., 2023 [47]	Ogata et al., 2023 [48]
Title and abstract	X			
Introduction				
Background/rationale	X	X	X	X
Objectives	X	X	X	X
Methods				
Study design	X		X	X
Setting	X	X	X	X
Participants	X	X	X	X
Variables	X	X	X	X
Data sources/measurement	X	X	X	X
Bias				
Study size				
Quantitative variables	X	X	X	X
Statistical methods	X	X	X	X
Results				
Participants				
Descriptive data	X	X	X	X
Outcome data	X	X	X	X
Main results	X	X	X	X
Other analyses	X	X	X	X
Discussion				
Key results	X	X	X	X
Limitations	X	X	X	X
Interpretation	X	X	X	X
Generalisability		X	X	X
Other information				
Funding	X	X	X	X
Total	18	17	19	18

**Table 2:** Systematic Review Center for Laboratory Animal Experimentation (SYRCLE) for animal studies

Type of bias	Domain	Issler et al., 2014 [25]	Patrício et al., 2020 [49]	Lo Iacono et al., 2021 [50]	Zhang et al., 2015 [51]	Song et al., 2019 [52]	Xie et al., 2019 [53]	Grieco et al., 2017 [54]
Selection bias	Sequence generation	No	Unclear	Unclear	Unclear	Yes	No	No
	Baseline characteristics	Yes	Yes	Yes	Yes	No	Yes	Yes
	Allocation concealment	Unclear	Unclear	Unclear	Unclear	Unclear	Unclear	Unclear
Performance bias	Random housing	Yes	Yes	Yes	Yes	Unclear	Yes	Yes
	Blinding	Unclear	Yes	Unclear	Yes	Unclear	Unclear	Unclear
Detection bias	Random outcome assessment	Unclear	Unclear	Unclear	Yes	Unclear	Unclear	Unclear
	Blinding	Yes	Yes	Yes	Yes	Unclear	Yes	Yes
Attrition bias	Incomplete outcome data	Unclear	Unclear	Unclear	Unclear	Yes	No	Unclear
Reporting bias	Selective outcome reporting	Unclear	Yes	Yes	Yes	Yes	Unclear	Yes
Other	Other sources of bias	Yes	Yes	Unclear	Yes	Yes	Unclear	Yes

Type of bias	Domain	Wan et al., 2018 [55]	Mingardi et al., 2021 [56]	Huang et al., 2021 [57]	Pan et al., 2015 [58]	Higuchi et al., 2016 [59]	Zheng et al., 2023 [60]	Guan et al., 2023 [61]
Selection bias	Sequence generation	Unclear	Unclear	No	Yes	No	Unclear	Unclear
	Baseline characteristics	Yes	Yes	Yes	No	Yes	Yes	Yes
	Allocation concealment	Unclear	Unclear	Unclear	Unclear	Unclear	Unclear	Unclear
Performance bias	Random housing	No	Yes	Yes	Unclear	Unclear	Yes	Unclear
	Blinding	No	Yes	Unclear	Unclear	Unclear	Unclear	Unclear
Detection bias	Random outcome assessment	Unclear	Yes	Unclear	Unclear	Unclear	Unclear	Unclear
	Blinding	Yes	Yes	Unclear	Yes	Yes	Unclear	Unclear
Attrition bias	Incomplete outcome data	Unclear	No	No	Yes	Unclear	Unclear	Unclear
Reporting bias	Selective outcome reporting	Yes	Yes	Yes	Yes	Yes	Yes	Unclear
Other	Other sources of bias	Yes	Yes	Yes	Yes	Yes	Yes	Yes

Results

**Figure 1:**
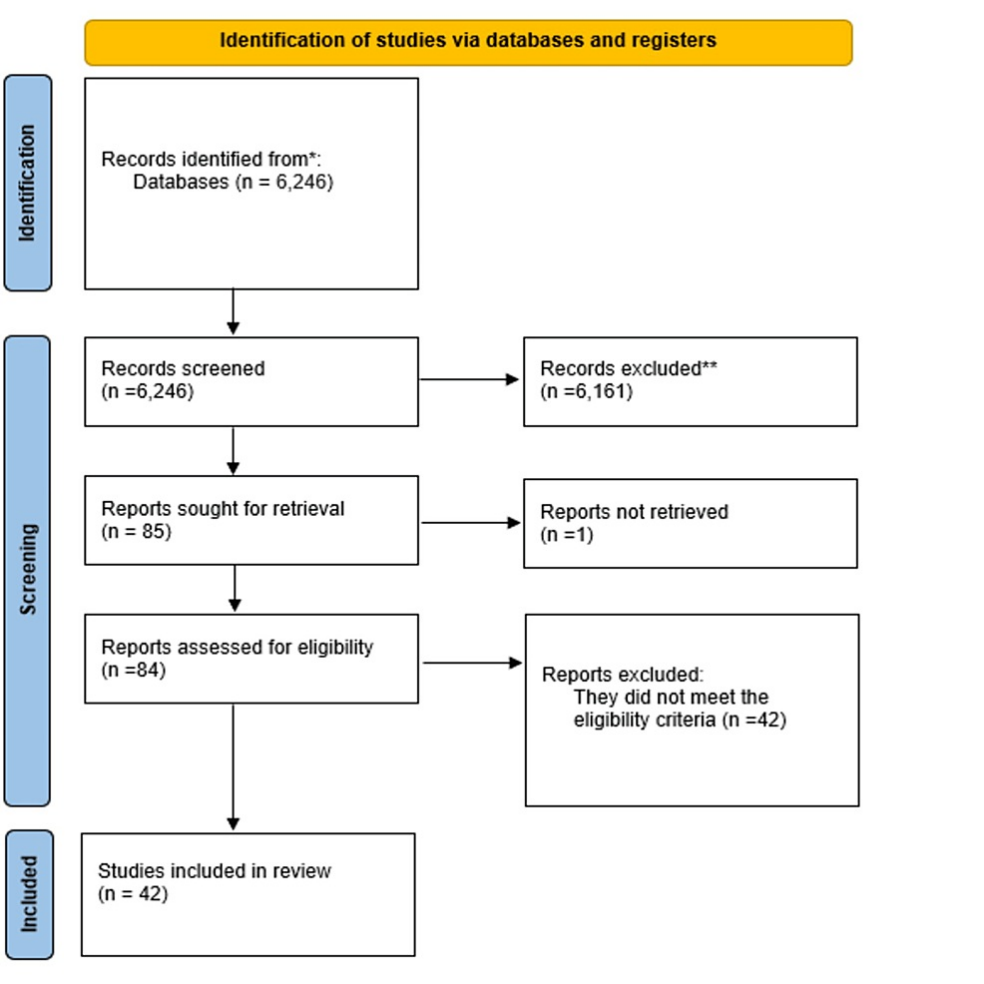
PRISMA flow diagram PRISMA: Preferred reporting items for systematic reviews and meta-analyses

The initial search of the databases returned 6,246 articles, and after reviewing the titles, 201 of these were selected for abstract perusal. A full reading was performed on the 84 studies selected based on the abstract, and 42 of these were included in the analysis and data extraction procedure (Figure 1): 15 studies were conducted in China; seven in Canada; four in Japan; three each in Italy, Taiwan, and Israel; two in Romania; and one each in Portugal, Ireland, France, Iran, and the United States. Two of the included studies evaluate two subpopulations: one includes humans and rodents [25] and the other humans and cell culture [14]. Thus, although when adding up the number of studies included in the tables there appears to be 44 studies, only 42 were actually included. The majority were human studies that used data from cohorts or clinical trials.

Human Beings

We found 28 studies comparing the miRNA expressions of people undergoing AD treatment (Table 3). The miRNA levels of patients were assessed following various therapeutic regimens. In 11 studies, participants were treated exclusively with selective serotonin reuptake inhibitors (SSRIs), with escitalopram/citalopram drugs used in eight of these.

**Table 3:** Summary of 28 studies investigating miRNA expression in humans after MDD treatment Abbreviations: miRNA - microRNA, MDD - major depressive disorder, MDE - major depressive episode, HDRS - Hamilton rating scale for depression, PBMCs - peripheral blood mononuclear cells, SIRT1 - sirtuin 1, SSRI - selective serotonin reuptake inhibitor, SNRI - serotonin-norepinephrine reuptake inhibitor, CBT - cognitive behavioral therapy, ECT - electroconvulsive therapy

Author/year	Population	Treatment	Sample	MicroRNAs in depression	MicroRNAs after treatment
Belzeaux et al. [13], 2017 (Canada)	MDE (n=106)	Citalopram	Blood		High levels miR-135 and miR-16 were associated with clinical improvement.
Lopez et al. [14], 2017 (Canada)	Discovery cohort MDD (n=258)	Duloxetine/placebo escitalopram or nortriptyline escitalopram	Blood		Decreased: miR-146a-5p, miR-146b-5p, miR-24-3p, miR-425-3p and miR-3074-5p; escitalopram responders: lower miR-146a-5p, miR-146b-5p and miR-24-3p levels
Bocchio-Chiavetto et al. [23], 2013 (Italy)	MDD treated (n=10)	Escitalopram	Blood		Increased: hsa-miR-130b, hsa-miR-505, hsa-miR-29b-2, hsa-miR-26b, hsa-miR-22, hsa-miR-26a, hsa-miR-494, hsa-let-7d, hsa-let-7g, hsa-let-7e, hsa-let-7f, hsa-miR-629, hsa-miR-606b, hsa-miR-103, hsa-miR-191, hsa-miR-128, hsa-miR-502-3p, hsa-miR-374b, hsa-miR-132, hsa-miR-30d, hsa-miR-500, has-miR-589, has-miR-183, hsa-miR-574-3p, hsa-miR-140-3p, hsa-miR-335, hsa-miR-361-5p); Reduced: miR-34c-5p and miR-770-5p
Homorogan et al. [24], 2021 (Romania)	MDD patients (n=11), healthy controls (n=11)	Escitalopram (10 mg)	Total plasma (TP), exosomes from plasma (EXO), exosmoes depleted plasma (EDP), peripheral nucleated blood cells (PNBCs)		MiR-494 was significantly differentially expressed in EDP
Issler et al. [25], 2014 (Israel)	MDD (n=77), MDD treated (n=77), healthy controls	Escitalopram	Blood	MiR-135a levels were robustly reduced in depressed patients compared to controls.	None difference was observed
Enatescu et al. [26], 2016 (Romania)	MDD treated (n=5)	Escitalopram	Blood		Increased: hsa-miR-1193, hsa-miR-3173-3p, hsa-miR-3154, hsa-miR-129-5p, hsa-miR-3661, hsa-miR-1287, hsa-miR-532-3p, hsa-miR-2278, hsa-miR-3150a-3p, hsa-miR-3909, hsa-miR-937, hsa-miR-676, hsa-miR-489, hsa-miR-637, hsa-miR-608, hsa-miR-4263, hsa-miR-382, hsa-miR-3691-5p, hsa-miR-375, hsa-miR-433, hsa-miR-1298, hsa-miR-1909, hsa-miR-1471. Reduced: hsa-miR-151-5p, hsa-miR-99b, hsa-miR-223, hsa-miR-181b, hsa-miR-26a, hsa-miR-744, hsa-miR-301b, hsa-miR-27a, hsa-miR-24, hsa-miR-146a, hsa-miR-126, hsa-miR-151-3p, hsa-let-7d, hsa-miR-221, hsa-miR-146b-5p, hsa-miR-125a-5p, hsa-miR-652
Saeedi et al. [27], 2021 (Canada)	MDD treated (n=40), healthy controls (n=20)	Escitalopram	Neuron-derived extracellular vesicles isolation from plasma		The combination of miR-21-5p, miR-30d-5p, miR-486-5p changes over treatment was associated with response (AUC=0.8254, specificity 84%, sensitivity 76%, P < 0,01)
Fang et al. [28] 2018 (China)	MDD treatment-free (drug naïve) (n=45) 14 treated later, MDD treated (n=32), healthy controls (n=32)	Citalopram	Blood		Decreased: miR-132, increased: miR-124
Wang et al. [29], 2018 (China)	MDD treated (n=68), healthy controls (n=42)	Citalopram	Blood	Increased: miR-155	Reduced: miR-155
Kuang et al. [30], 2018 (China)	MDD treated (n=84), healthy controls (n=78)	Paroxetine	Blood	Increased: miR-34a-5p and mi-221-3p	Increased: miR-451a, decreased: miR-34a-5p and miR-221-3p. MiR-451a was negatively correlated with the HDRS score. MiR-34a-5p and miR-221-3p were positively correlated with HDRS scores
Lin et al. [31],2018 (Taiwan)	MDD treated (n=33)	SSRI and SNRI	Blood		Increased: miR-183 miR-212 and miR-16
Fiori et al. [32], 2017 (Canada)	Replication cohort 1 (n=61), replication cohort 2 (n=158)	Escitalopram, desvenlafaxine	Blood	Responders: lower basal levels of miR-1202	Responders: higher elevation miR-1202
Kato et al. [33], 2022 (Japan)	MDD treated (n=92)	Paroxetine, sertraline, mirtazapine	Blood		SSRI: 228 miRNAs were significantly correlated (miR-483.5p; miR-3151.5p). Mirtazapine: 9 miRNAs were significantly correlated (miR-483-3p)
Kim et al. [34], 2019 (Canada)	Cohort A MDD treated (n=212), healthy controls (n=238), cohort B MDD treated (n=181), healthy controls (n=180), cohort C MDD treated (n=230), healthy controls (n=245)	Duloxetine	Blood		Remitted treatment: high levels Hsa-miR-16-5p, high levels in responders: hsa-miR-146a-5p, hsa-miR-21-5p, and hsa-miR-30b-5p. Hsa-miR-23a-3p high levels in responders: Hsa-miR-21-5p, hsa-miR-144-3p, hsa-let-7c-5p, and hsa-miR-30c-5p
Marshe et al. [35], 2020 (Canada)	T0 (n=311 older adults >60y), MDD T12 top 100 remitters and bottom 100 non-remitters, final sample (n=184)	Venlafaxine	Blood		Remitters: nominally significant decrease levels of miR-1202 and miR-425-3p
Gururajan et al. [36], 2016 (Ireland)	MDD treated (n=16), healthy controls (n=20)	Ketamine	Blood		No difference in miRNA expression levels was observed in the ketamine group
Belzeaux et al. [37], 2012 (France)	MDE treated (n=16), healthy controls (n=13)	SSRI, SNRI, other antidepressant, lithium, atypical antipsychotics and ECT	Blood		Reduced: miR-200c, increased: miR-107, miR-342-5p, and let-7b
Qiao-li et al. [38], 2014 (China)	MDD treated (n=81), healthy controls (n=46)	Venlafaxine, sertraline and mirtazapine	Blood	Increased: miR-26b, miR-4743, miR4498, miR-4485, miR-1972	
He et al. [39], 2016 (China)	MDD treated (n=32), healthy controls (n=30)	Venlafaxine, paroxetine, fluoxetine, escitalopram, duloxetine, sertraline and mirtazapine	Blood	Increased: miR-124	Reduced: miR-124
Hung et al. [40],2019 (Taiwan)	MDD treated (n=84), healthy controls (n=43)	Escitalopram, fluoxetine, paroxetine, sertraline, duloxetine, venlafaxine, bupropion, mirtazapine, and agomelatine.	Blood - PBMCs	Reduced: let-7e, miR-21-5p, miR-146a, and miR-155 in PBMCs	Increased: let-7e, miR-223, miR-146a, and miR-155; increased: let-7e, miR-223, miR-145, and miR-155 in remittent
Qi et al. [41], 2020 (Canada)	MDD treated (n=140), healthy controls (n=28)		Blood		Cluster 1: hsa-miR-5701 higher levels in responders. Cluster 2: hsa-let-7b-3p and hsa-miR-130b-3p higher levels in responders
Hung et al. [42], 2021 (Taiwan)	MDD treated (n=39), MDD (n=52), healthy controls (n=31)	Escitalopram Fluoxetine Paroxetine Sertraline Duloxetine Venlafaxine Bupropion Mirtazapine Agomelatine	Serum exosomes	Remitted patients: Reduced: let-7e, miR-21-5p, miR-145, miR-146a, and miR-155.	Remitted patients Increased: let-7e, miR-21-5p, miR-145, miR-146a, and miR-155
Liu et al. [43], 2021 (China)	MDD treated (n=21) MDD (n=5), healthy controls (n=5)	Escitalopram Paroxetine Venlafaxine Fluoxetine Sertraline mirtazapine	Blood	Reduced: miR-324p and miR-10a	Unaltered
Song et al. [44], 2022 (China)	MDD treated (n=29) MDD (n=80)	Sertraline	PBMCs	Increased: miR-4485 score of HAMD was negatively correlated with the expression level of miRNA4485	Increased: miRNA-4485 miRNA-4485 was positively correlated with the improvement of depressive symptoms
Emami et al. [45], 2023 (Iran)	MDD (n=50), healthy volunteers (n=20)	Sertraline citalopram	Plasma	Reduced: miR-16 Increased: miR-132 and miR-124	Increased: miR-16, sertraline reduced: miR-132 and miR-124
Wang et al. [46], 2023 (China)	MDD (n=48), healthy controls (n=50)	It doesn’t inform	Whole blood	Reduced: miR-16-2, Negatively correlated with HAMD-17 and HAMA-14 scores (AUC=0.806, 95 % CI: 0.721–0.891)	Patients with higher miR-16-2 expression levels were more likely to exhibit a higher rate of score reduction in HAMD-17 than the low-expression group in the short-term follow-up
Funatsuki et al. [47], 2023 (Japan)	MDD treated (n=46)	Mirtazapine SSRIs	Plasma		In remitted patients to mirtazapine, reduced: miR-1237-5p (AUC=0.85, 95% confidence interval=0.72–0.97)
Ogata et al. [48], 2023 (Japan)	MDD (n=65)	Mirtazapine SSRIs	Plasma		SSRI remitters: lower basal levels of hsa-miR-4707-3p, mirtazapine remitters higher basal expression of hsa-miR-6068, and it was inversely correlated with HAM-D 17

In2013, Bocchio-Chiavetto et al. [23] determined that, after 12 weeks of treatment with escitalopram, 28 miRNAs became overexpressed, with fold changes ranging from 2.07 to 4.68, whereas only two miRNAs were highly downregulated (miR-34c-5p and miR-770-5p), with fold decreases of 2.03 × 10-2 and 1.69 × 10-2, respectively [23]. Homorogan et al. [24] found that miR-494 was differentially expressed in exosome-depleted plasma after 12 weeks of treatment with escitalopram. Additionally, in a study comparing MDD patients with healthy controls, the MDD group presented lower miR-135a levels in the blood samples taken, and after 12 weeks of escitalopram treatment or cognitive behavioral therapy (CBT), patients undergoing CBT had an increase in miR-135a expression [25]. These effects were not significant in the tests with miR-16 [25]. Furthermore, the brain miR-135a and miR-16 levels of suicide victims were significantly lower than those of the controls, specifically in the dorsal raphe and raphe magnus subnuclei [25]. Enatescu et al. [26] assessed the peripheral expression changes of 222 miRNAs in five MDD patients treated with escitalopram for 12 weeks and found that 23 were upregulated and that many of these presented a greater than 2.5 times increases, while 17 of the miRNAs were downregulated [26].

Saeedi et al. [27] found that the combination of changes in miR-21-5p, miR-30d-5p, and miR-486-5p with escitalopram treatment was associated with treatment response (area under the curve (AUC) = 0.8254, specificity of 84%, sensitivity of 76%, P < 0.01). This was the only study that analyzed the specificity and sensitivity of a cluster of microRNAs and highlighted them as good biomarkers of the response to AD treatment with escitalopram. Moreover, when comparing the miR-132 and miR-124 plasma changes among MDD patients who were drug naïve, those who used citalopram, and a control group of individuals without depression, those authors found that miR-132 levels were 2.4 times higher in the drug-naïve group when compared to the control, while MDD patients undergoing citalopram had similar miR-132 levels to those of the control group [28]. However, both MDD groups showed higher miR-124 plasma levels than the control group (1.8-fold and fourfold in the drug-naïve and citalopram-treated groups, respectively) [28]. After two months of treatment with citalopram, a decreasing trend in plasma miR-132 and an increase in miR-124 was detected in the 14 drug-naïve patients tested [28]. Another study indicated that miR-155 levels were downregulated after treatment with citalopram [29], whereas both cellular and serum levels of miR-155 were found to be significantly upregulated among MDD patients compared to those of healthy controls [29]. Kuang et al. [30] found decreased levels of miR-451, and higher miR-34a-5p and miR-221-3p levels in depressed patients pre- and post-paroxetine treatment compared to the healthy control group. After treatment with paroxetine, miR-451a levels increased, although both miR-34a-5p and miR-221-3p levels decreased significantly when compared to the basal levels [30].

Participants treated solely with SSRIs presented increased levels of miR-16, while patients treated with either SSRIs or serotonin and norepinephrine reuptake inhibitors (SNRI) exhibited increased expressions of miR-183 and miR-212 [31]. Low baseline levels of miR1202 were associated with improved therapeutic responses to SSRIs and SNRIs; MDD patients who responded to AD treatment had lower basal levels of miR-1202, and these levels increased after the drug was administered [32]. Moreover, the prediction analyses of nonresponse found a sensitivity of 91.7%, a specificity of 67.7%, and an AUC = 0.812 [32]. Among patients treated with an SSRI (paroxetine or sertraline), a significant correlation was identified two weeks after treatment initiation between 228 miRNAs (especially miR-483-5p and miR-3151-5p) and depression severity among patients treated with mirtazapine, with the strongest association found with miR-483-3p [33].

Three studies assessed miRNA levels in patients treated with duloxetine, venlafaxine, or ketamine. For patients treated with duloxetine, baseline levels of hsa-miR-23a-3p were associated with a remission of depressive symptoms [34]. Belzeaux et al. [13] reported elevated baseline levels of miR-135a-5p and miR-16 and improved treatment response in patients administered citalopram. Patients whose depressive symptoms abated had nominally significant decreases in miR-1202 and miR-425-3p levels [35]. Among the patients who received ketamine, there were no differences in miRNA expression when compared to the control group [36]. Other studies evaluated patients who received different ADs. Belzeaux et al. [37] found that, after the treatment of patients experiencing a major depressive episode, miR-107, miR-342-5p, and let-7b levels were upregulated, while miR-200c expression was downregulated [37]. Depressed patients showed significantly higher expressions of miR-26b, miR-4743, miR-4498, miR-4485, and miR-1972 before treatment [38].

Various studies have investigated the basal characteristics of patients in the search for treatment response predictors and the untreated MDD miRNA profile. Peripheral blood mononuclear cells (PBMCs) showed elevated levels of miR124 in patients with depression, and a reduction in these levels was associated with improved treatment responses [39]. After adjustments for age, sex, smoking status, and body mass index (BMI), the levels of let-7e, miR-21-5p, miR-146a, and miR-155 were significantly lower in PBMCs isolated from baseline MDD patients [40], while let-7e and miR-146a were negatively correlated with the Hamilton Depression Rating Scale (HAMD)-17 score after treatment, and miR-155 was positively correlated with this value [40]. Qi et al. [41] used machine-learning analyses of miRNA to reveal high hsa-miR-5701 levels among responders in one cluster and high hsa-let-7b-3p and hsa-miR-130b-3p levels in a second cluster [41]. Reduced levels of miR-146b-5p, miR-24-3p, and miR-425 were associated with better responses to treatment in both human and animal models [14]. Hung et al. [42] showed that patients who achieved remission after treatment with the different antidepressants had lower basal levels of let-7e, miR-21-5p, miR-145, miR-146a, and exosomal miR-155 compared to those of healthy controls, which was followed by a notable increase in these levels post-treatment. Although most studies showed changes in miRNA levels after treatment, Liu et al. [43] found reduced baseline levels of miR-324b and mir-10a in depressed patients when compared to healthy controls that were unchanged after eight weeks of treatment with classic antidepressants. The sertraline treatment reduced the high miR-4485 levels shown in MDD patients that were negatively correlated with HAMD scores [44].

Two recently published studies have identified decreased levels of miR-16 in patients diagnosed with MDD, with one study demonstrating an increase in these levels following treatment with sertraline or escitalopram [45,46]. Sertraline, but not escitalopram, was found to significantly reduce the levels of miR-132 and miR-124, both of which were elevated in MDD patients [45]. Furthermore, according to Wang et al. [46], it was observed that miR-16-2 exhibited a negative correlation with both HAMD-17 and HAMA-14 scores (AUC = 0.806, 95% CI = 0.721-0.891) [46]. Patients displaying higher expression levels of miR-16-2 were more inclined to experience a greater reduction in HAMD-17 scores during short-term follow-up compared to the low-expression group [46].

The remaining two studies discovered decreased levels of miR-1237-5p in patients who experienced symptom remission with mirtazapine (AUC = 0.85, 95% CI = 0.72-0.97), along with elevated basal expression of hsa-miR-6068 [47,48]. Moreover, one of these studies noted a decrease in basal levels of hsa-miR-4707-3p in remitters to SSRIs [48].

Animal Models

We found 14 studies comparing the miRNA expressions of rodents undergoing AD treatment (Table 4). Three SSRI studies reported diverse miRNA profiles and variable treatment response patterns among the animal samples. Patricio et al. [49] investigated changes in miR-409-5p and miR-411-5p levels in brain regions linked to depression in rats undergoing chronic mild stress (CMS). The rats received treatment with either fluoxetine or a saline solution [49]. The stress-exposed animals presented significantly increased levels of miR-409-5p in the nucleus accumbens that were not reversed with fluoxetine treatment [49]. In the dorsal dentate gyrus, chronic fluoxetine use resulted in increased levels of miR-411-5p; therefore, miR-411-5p levels were significantly decreased in the blood of fluoxetine-treated rats [49]. The genetic deletion of miR-34s impairs the effect of chronic fluoxetine on the behavioral responses of rats to an acute threat [50]. Chronic fluoxetine treatment increased the levels of miR-34a in WT mice, but this was not observed with acute administration of this compound [50].

**Table 4:** Summary of 14 studies investigating miRNA expression in animal models after AD use Abbreviations: CT - non-stressed control, CMS - chronic mild stress, CUS - chronic unpredictable stress, vDG - ventral dentate gyrus, NAc - nucleus accumbens, dDG - dorsal dentate gyrus, CUMS - chronic unpredictable mild stress, PFC - prefrontal cortex, miRNA - microRNA

Author/year	Population	Treatment	Sample	MicroRNAs in depression	MicroRNAs after treatment
Issler et al. [25], 2014 (Israel)	Mice - social defeat, mice treated	Tricyclic, imipramine, fluoxetine or reboxetine	Raphe nuclei, blood	Reduced: miR-135a in blood	Imipramine and fluoxetine, increased: miR-135a in raphe nuclei SSRI, increased: miR-135a in blood
Patricio et al. [49], 2020 (Portugal)	Non-stressed control (N=10-12), CMS group (N=10-12), CMS+fluoxetine (N=10-12)	Fluoxetine	Brain, blood	Increased: miRNA-409-5p in the NAc	Increased: miR-409-5p in vDG, reduced: miR-411-5p in the dDG, reduced: miR-409-5p and miR-411-5p in blood
Lo Iacono et al. [50], 2021 (Italy)	Knockout (TKO) mice, wild-type (WT) mice	Fluoxetine	Dorsal raphe		Chronic treatment, increased: miR-34a in WT mice
Zhang et al. [51], 2015 (China)	Normal control (NOR, n=15), maternal deprivation (MD, n=12), chronic unpredictable stress (CUS, n=11), MD with CUS (MD+CUS, n=11)	Escitalopram	NAc and the striatum	Increased: miR-326 in NAc, decreased: miR-9 in NAc and striatum, decreased: miR-326 in striatum	Reverted the miR-326 to normal levels in NAc
Song et al. [52], 2019 (China)	Control group (N=8), CUMS group + escitalopram (N=8), CUMS not treated (N=8)	Escitalopram	Nac	Reduced: miR-10b-5p, increased: miR- 214-3p	Increased: miR-10b-5p, reduced: miR-214-3p
Xie et al. [53], 2019 (China)	Controls (N=8), imipramine (N=8), fluoxetine (N=8)	Imipramine, fluoxetine, reboxetine	Dorsal raphe nucleus	Reduced: miR-26	Imipramine and fluoxetine, increased: miR-26a-2
Grieco et al. [54], 2017 (USA)	C57BL/6 wild-type and homozygous GSK3α/β 21A/21A/9A/9A knockin mice	Ketamine, 2,3-dihdroxyl-6-nitro-7-sulfamoylbenzo(f)quinoxaline-2, 3-dione or fluoxetine	Hippocampus and PFC		Ketamine Increased: miR-764-5p, miR-1912-3p, miR-1264-3p, miR-1298-5p, and miR-448-3p in the hippocampus
Wan et al. [55], 2018 (China)	CUMS Controls	Ketamine	PFC	Reduced: miR-29b-3p	Increased: miR-29b-3p
Mingardi et al. [56], 2021 (Italy)	72 Sprague-Dawley male rats, divided according to vulnerability or resilience to CMS, and CMS-vulnerable rats were evenly randomized to ketamine or saline	Racemic ketamine (10 mg/kg) - single dose	Hipoccampus (brain tissue and cell culture)		Ketamine restored original miR-9-5p levels, which was correlated with higher sucrose preference/less anhedonic phenotype in rats, and increased dendritic length in vitro
Huang et al. [57], 2021 (China)	Control CSDS-resilient mice and saline- or ketamine administered CSDS-susceptible mice (n = 8 per group)	Ketamine (10 mg/kg), aline	Hippocampus and cortex	Reduced: miR-98-5p	Increased: miR-98-5p
Pan et al. [58],2015 (China)	30 animals (mice): Control group Model group Duloxetine group CUMS	Duloxetine	Frontal lobe and hippocampus	Reduced: miR-132 and miR-18a in the hippocampus, increased: miR-134 and miR-124a	Increased: miR-132 and miR-18a in the hippocampus, decreased: miR-134 and miR-124a
Higuchi et al. [59], 2016 (Japan)	BALB mice CUMS, control	Imipramine	Hippocampus	Reduced: miR-124-1 and miR-29a	These effects were blocked by imipramine
Zeng et al. [60], 2023 (China)	Adult male BALB/c mice (18–22 g, 8 weeks old) CUMS	Fluoxetine	Hippocampus	Increased: miR-124	Reduced: miR-124 levels
Guan et al. [61], 2023 (China)	Adult male C57BL6/J mice	Venlafaxine	Hippocampus	Increased: miR-204-5p	Reduced: miR-204-5p

Another study investigated the changes in the expression of miR-326 and miR-9 [51]. Adult rats exposed to chronic unpredictable stress (CUS) and maternal deprivation had lower levels of miR-9 in the nucleus accumbens and striatum, and these levels did not rise after escitalopram treatment [51]. In the nucleus accumbens, the altered expression of miR-9 was negatively correlated with an overexpression of the dopamine receptor D2 (DRD2) protein, which appears to be involved in chronic stress and early-life adversity [51]. The expression of miR-326 was increased in the nucleus accumbens and decreased in the striatum of rats exposed to CUS [51]. In addition, animals with maternal deprivation showed increased levels of miR-326 in the striatum [51]. In contrast to the miR-9 findings, miR-326 levels returned to normal after escitalopram treatment [51]. In another study, escitalopram treatment reversed miRNA expression alterations in rats after chronic unpredictable mild stress (CUMS) [52]. Although those authors found 18 miRNA alterations, they suggested that miR-10b-5p and miR-214-3p had important contributions to the miRNA network of the nucleus accumbens since the expression of these miRNAs differed significantly between the stressed group and the control and between the treated group and the control [52].

The miRNAs related to serotoninergic networks were influenced by AD treatments in some studies. A potential regulator of serotoninergic activity, miR-135, was increased after AD treatment [25]. High levels of miR-135a appear to diminish depression-like behaviors after stress exposure, whereas its absence reduces the response to ADs [25]. A study using transgenic mice suggested that miR-26a-2 levels are involved in the serotoninergic network, given that ADs upregulate the expression of this miRNA in the dorsal raphe nucleus [53].

The influence of ketamine on miRNA expression was evaluated, and one study reported variable changes in the expression of miRNA serotonergic (5HT)-2C-receptor (5HTR2C) clusters in mouse hippocampi [54]. Following 24 hours of ketamine infusion, miR-764-5p, miR-1912-3p, miR-1264-3p, miR-1298-5p, and miR-448-3p were upregulated [54], with miR-448-3p showing the greatest increase and poorest AD response when it was inhibited [54]. These changes were not replicated in the prefrontal cortex [54]. The same miRNA changes were not observed in mice receiving fluoxetine, which suggests that these are part of a ketamine response and not just a typical AD response [54]. In addition, miR-29b-3b appears to be involved in the AD effect of ketamine. A study found that this miRNA level was reduced in the prefrontal cortex of rats after CUMS; however, after ketamine treatment, the miR-29b-3b levels were reestablished [55].

Another study found that the administration of a single dose of ketamine in CMS rats restored the original miR-9-5p levels, and this increase was correlated with higher sucrose preference/less anhedonic phenotype [56]. In addition, Huang et al. [57] explored the antidepressant effect of ketamine in an animal model of depression and observed a reduction in miR-98-5p levels in the prefrontal cortex and hippocampus after treatment.

In a study with mice undergoing CUMS, duloxetine was found to enhance the expression of miR-18a in the frontal lobe, as well as upregulate miR-132 and miR-18a and downregulate miR-134 and miR-124a in the hippocampus [58].

Higuchi et al. [59] found lower levels of miR-124 in the hippocampus of mice subjected to chronic stress. The upregulation of miR-124 in the hippocampus increased resilience in mice, while its downregulation enhanced depression-like behaviors [59]. On the other hand, Zeng et al. [60] found an increased level of miR-124 in the hippocampus of mice with symptoms such as depression, and the fluoxetine administration reduced these levels. Guan et al. [61] investigated the levels of miR-miR-204-5p in rats with a depressive-like phenotype and possible changes caused by treatment with velanfaxine. The group found higher levels of miR-204-5p in depression, and the treatment reduced these levels [61].

Cell Cultures

We included four cell culture studies targeting different miRNAs and a variety of sample cultures (Tables 5-6). Three of these used paroxetine and one used duloxetine.

**Table 5:** Summary of four studies investigating miRNA expression in human cell cultures after ADs use Abbreviations: LCLs - human lymphoblastoid cell lines, miRNA - microRNA

Author/year	Population	Treatment	Sample	MicroRNAs after treatment
Lopez et al. [14], 2017 (Canada)	Human neural progenitor cells	Duloxetine	Human neural progenitor cells	Reduced: miR-146a-5p, miR-146b-5p, miR-24-3p, and miR-425-3p
Oved et al. [62], 2012 (Israel)	LCLs from 80 healthy adult female donors	Paroxetine	LCLs	MiR-151-3p: increased sensitivity to paroxetine MiR-212, miR-132, miR-30b*, let-7b and let-7c also differed between paroxetine and control groups
Angelucci et al. [63], 2011 (Italy)	Cell cultures and control cultures	Paroxetine	Human glioblastoma-astrocytoma;	6 h and 12 h of incubation Increased: miR-30a 24h of incubation: miR-30a expression returned to levels comparable to untreated cells
Oved et al. [64], 2013 (Israel)	Cell cultures and control cultures	1mM paroxetine for 21 days X phosphate-buffered saline	Cell cultures (human)	Reduced: miR-221 and miR-22

**Table 6:** Most consistently expressed miRNA Abbreviations: miRNA - microRNA

MicroRNA	Expression in depression	Expression after treatment	Samples	Treatment	Remission marker	Authors
MiR-1202	Low Levels	Elevation	Human blood	Escitalopram, desvenlafaxina, venlafaxina	Yes	Fiori et al. [32], 2017; Marshe et al. [35], 2020
MiR-135	Low Levels	Elevation	Human blood, raphe nuclei – mice	Citalopram, escitalopram	Yes	Belzeaux et al. [13],2016; Issler O et al. [25], 2014
MiR-124	High Levels	Reduction	Human blood, frontal lobe, and hippocampus (mice)	Venlafaxine, paroxetine, fluoxetine, escitalopram, duloxetine, sertraline, mirtazapine, citalopram, imipramine	Yes	He et al. [39], 2016; Fang et al. [28], 2018; Pan et al. [58], 2015; Higuchi et al. [59], 2016
MiR-16	Low Levels	Elevation	Human blood	Citalopram, duloxetine	Yes	Belzeaux et al. [13], 2016; Kim et al. [34], 2019; Lin et al. [31], 2018

The exposure of human lymphoblastoid cell lines to paroxetine for three days increased the expression of miR-212, miR-132, miR-30b, let-7b, and let-7c [62], and its incubation for 21 days decreased the expression of miR-221 and miR-22.55. The first study identified a correlation that mainly concerned the miR-151-3p basal levels, which were higher in cells that were sensitive to the paroxetine treatment [62]. Another study by the same group primarily showed downregulation of miR-221 and miR-222 after treatment [63]. The third study using paroxetine analyzed glioblastoma-astrocytoma cell line U87 and found a significant upregulation of miR-30a levels beginning six hours after treatment, which lasted for 12 hours after treatment [64]. The final study used duloxetine as the treatment, and this was previously discussed in the human studies section of this article [14]. Those authors confirmed their previous findings on human and rodent studies in an in vitro analysis using human neural progenitor cells, with reduced levels of miR-146a-5p, miR-146b-5p, miR-24-3p, and miR-425-3p in the cell culture [14].

Discussion

We found several studies that evaluated miRNA expression in humans, animals, and cell cultures, both before and after receiving ADs. Different classes of AD were used, as well as a broad profile of expressed miRNAs.

The studies revealed altered levels of specific miRNAs in patients with MDD before and after receiving treatment, which indicates that miRNAs could be involved in the pathophysiology of MDD and response to ADs and be potential biomarkers for MDD diagnosis and therapeutic response. The studies evaluated were heterogeneous, with no pattern of change observed in the miRNAs; therefore, more robust studies with larger samples and consistent methodologies are required.

Most of the microRNAs investigated in the studies are suggested to be involved in the mechanisms of neuroplasticity, such as in the expression of brain-derived neurotrophic factor (BDNF), which is an important neurotrophin that is possibly involved in MDD pathophysiology and treatment response to ADs such as miR-1202, miR-124, miR-16, and miR-135 [59,65]. Wibrand et al. [65] reported that the overexpression of miR-34a was linked to a reduction in the BDNF.

Another microRNA that may be important for the pathophysiology of depression and AD response is miR-124 [59], which could have a central role in neurogenesis and neuronal function, as well as in regulating proliferation, growth, and apoptosis of the central nervous system (CNS) [28,39,66]. Mir-124 has also been shown to negatively affect BDNF expression [39,67]. Shi et al. [68] provided evidence supporting the role of miR-124 in depression-induced mice, which had increased depression‐like behaviors and higher miR‐124 expression levels in the hippocampus than the control mouse. In addition, those authors found that the genetic knockdown of hippocampal miR-124 exerted significant antidepressant-like effects that were mediated by the promotion of BDNF biosynthesis. These results corroborate the findings presented in other articles regarding the importance of miR-124 in the physiopathology of depression and antidepressant response.

Mir-132 has been linked to important and critical CNS functions, including neurogenesis and synaptic plasticity in different brain regions [28,69]. MiR-212, miR-30b, let-7b, and let-7c have been identified as relevant to the neuroplasticity process, while miR-16, miR-183, miR-212, and miR-221-3p are related to BDNF expression [30,31,62,63].

Lopez et al. [14] proposed a mechanism of action for miR-146a/b-5, miR-425-3p, and miR-24-3p as regulators of the MAPK and Wnt pathways based on the results of studies conducted on humans and cell cultures. These miRNAs regulate a range of genes involved in the signaling process of the two pathways, in addition to interfering with the BDNF pathway. The genes regulated by these miRNAs have been linked to MDD and AD response. Other relevant microRNAs are miR-34c-5p and miR-34a-5p, which are suggested to be related to stress response and neuroplasticity [23,30], while the genetic deletion of the miR-34s can hinder the antidepressant effect [50].

MiR-135a is involved in the metabolism and level of 5-HT, whereby it is an important regulator for maintaining serotonergic tone to centrally favor the AD response [25]. MiR-135a-3p is involved in the modulation of the glutamatergic system and stress response, although its association with these processes is unclear [13,35].

Other microRNAs, such as let-7e, miR-21-5p, miR-146a, and miR-155, have been related to inflammatory processes that are associated with important pathways for the pathophysiology of MDD. In addition, two studies focusing on brain tissue miRNAs from MDD patients have identified the role of miR-1202 in regulating the glutamate receptor, which is a new pathway that is proposed to be involved in MDD. MiR-1202 is a primate-specific miRNA that is associated with the pathophysiology of MDD and its responsiveness to SSRIs [70]. The potential of miRNAs as biomarkers for response to treatment has been indicated [71]. MiR-1202 regulates metabotropic glutamate receptor 4 (GRM4), which is a proposed target glutamatergic receptor for the development of ADs [70]. AD treatment was shown to increase miR-1202 levels and decrease GRM4 levels [70]. Initial miR-1202 expression was lower in responders to ADs, while non-responders demonstrated no change in miR-1202 expression compared to healthy individuals [70].

Limitations, implications, and future directions 

The limitations of our study include the diversity of the populations studied (humans, animal models, and cell cultures), the wide range of ADs used, and the large number of miRNAs identified. Although a wide variety of miRNAs exist, there is little replication of studies that investigate the same molecules from different perspectives. Most studies are limited to evaluating the change of miRNAs in AD treatments, without assessing or describing the related action mechanisms. These aspects complicate meta-analyses; therefore, a discussion and understanding of the basic aspects of the pathophysiology of depression are limited. Although the literature has established a relationship between circulating miRNAs and MDD, it is important to understand the targets of these miRNAs using in vitro, in vivo, and in silico models.

The findings of this systematic review reveal that miRNAs have been linked to MDD and AD treatment. This knowledge can contribute to the understanding of depression etiology and antidepressant action. Based on these findings, miRNA expression can be used to direct the treatment of MDD [72]. Thus, this is the first review to explore the changes in miRNAs after AD exposure in three different populations (cell cultures, animal models, and human studies).

The studies included in this review assessed specific miRNAs that were associated with MDD and AD response. They provide evidence that specific miRNAs can act as biomarkers; however, the clinical utility of miRNAs as biomarkers of MDD and responses to ADs needs to be further investigated. Studies conducted in more homogeneous populations are recommended.

## Conclusions

The results of our systematic review demonstrated that certain microRNAs are altered in patients with MDD and can be modified with AD treatment, corroborating their potential as diagnostic and response biomarkers. Understanding the pathophysiology of MDD can improve the therapeutic options for this condition. Considering the heterogeneity of miRNAs across the studies, further investigation is required to evaluate the expressions of miRNAs in MDD and their changes with treatment to confirm these findings and provide a comprehensive explanation of the involved pathways. Existing evidence supports the hypothesis that miRNAs could have a place in the future of personalized psychiatry.
